# Long-term effectiveness and safety of vedolizumab in ulcerative colitis: a real-world retrospective cohort study over 2 years

**DOI:** 10.3389/fmed.2026.1795200

**Published:** 2026-05-18

**Authors:** Peipei Zhang, Xin Yu, Shengfu He, Jing Guan, Yumei Wu, Yuanyuan Zhao, Jing Hu, Qiuyuan Liu, Juan Wu, Qiao Mei, Wei Han

**Affiliations:** 1 Department of Gastroenterology, The First Affiliated Hospital of Anhui Medical University, Hefei, China; 2 Department of Gastrointestinal Surgery, The First Affiliated Hospital of Anhui Medical University, Hefei, China

**Keywords:** biologic therapy, long-term outcomes, real-world evidence, ulcerative colitis, vedolizumab

## Abstract

**Background:**

Vedolizumab (VDZ), a gut-selective anti-integrin therapy, has demonstrated efficacy in ulcerative colitis (UC) clinical trials, but real-world long-term data from Asia, particularly from Chinese populations, remain limited. Therefore, this study aimed to evaluate the long-term effectiveness and safety of vedolizumab in Chinese patients with UC in a real-world setting over a 2-year period.

**Methods:**

In this retrospective cohort study conducted at a tertiary hospital in China, patients aged ≥18 years old who received VDZ for UC between December 2020 and August 2023 were enrolled. Information on the clinical characteristics, prior/concomitant treatment, and safety during the observation period was collected.

**Results:**

The data obtained from 112 patients (males, *n* = 62) were analyzed. The median age at VDZ induction was 40.37 years old. VDZ was indicated as the first biologic in 85.71% of patients. Clinical remission rates at weeks 14, 54, and 104 were 61.61, 66.96, and 58.93%, respectively. VDZ persistence was 78.57% at 1 year and 61.61% at 2 years. Concomitant use of 5-ASA or steroids was associated with poorer drug persistence (HR = 3.13 and 5.03, *p* < 0.01). Clinical response at week 14 predicted sustained remission (OR = 6.07, *p* < 0.001). Nine adverse drug reactions (ADRs) were noted, none severe.

**Conclusion:**

This real-world study confirms vedolizumab’s sustained effectiveness in UC, with durable remission and excellent safety over 2 years. The findings support its position in treatment algorithms and highlight the value of early response assessment in guiding therapeutic decisions.

## 1 Introduction

Ulcerative colitis (UC) represents a chronic, relapsing inflammatory bowel disease characterized by diffuse mucosal inflammation of the colon, leading to debilitating symptoms including bloody diarrhea, abdominal pain, and systemic complications (1). While conventional therapies comprising 5-aminosalicylates, corticosteroids, and immunomodulators maintain foundational importance in management strategies (2), a substantial proportion of patients experience refractory disease, steroid dependence, or treatment-limiting adverse events. The introduction of biologic agents has substantially transformed UC management, providing targeted mechanisms to modify disease progression and enhance patient outcomes (3).

Vedolizumab, a humanized monoclonal antibody selectively targeting α4β7 integrin, inhibits lymphocyte trafficking to the gut microenvironment, thereby establishing gut-specific immunomodulation without systemic immunosuppression (4, 5). Pivotal phase 3 clinical trials, notably GEMINI 1 (NCT00783718), established its efficacy and safety profile in moderate-to-severe UC, demonstrating clinical remission rates of 44.3% at 52 weeks (4). The subsequent long-term safety (LTS) study (phase 3 extension, NCT00790933) further confirmed that clinical remission and mucosal healing were sustained through 152 weeks of continuous therapy, with no increased risk of serious infections or malignancy (6). More recently, the phase 3 VISIBLE 1 RCT (NCT02611830) established the non-inferiority of subcutaneous vedolizumab to intravenous formulation in maintaining clinical remission at week 52, supporting its long-term use with enhanced convenience (7). Collectively, these RCT data position vedolizumab as a reliable long-term therapeutic option for UC, offering sustained steroid-free remission and mucosal healing with a consistent safety profile (8, 9). However, it is important to note that pivotal trials such as GEMINI 1 and VISIBLE 1 predominantly enrolled Caucasian populations. Furthermore, randomized controlled trials inherently enroll homogeneous populations under stringent protocols, potentially constraining generalizability to real-world clinical settings where patients exhibit greater diversity in disease severity, comorbidities, and prior treatment failures. Beyond comparing effectiveness between specific medications, understanding which treatment strategy proves more appropriate for achieving favorable, long-term, patient-centered outcomes holds greater clinical relevance. This perspective intrinsically accommodates subsequent real-world decisions concerning treatment optimization, including switching to alternative therapies following first-line treatment failure or intolerance, and therapeutic sequencing. Although RCT extensions and registry studies offer valuable insights, real-world evidence remains indispensable for evaluating vedolizumab’s long-term effectiveness and durability while optimizing treatment strategies across diverse patient populations, particularly in Asian cohorts where data are scarce.

Therefore, this study was designed to assess the long-term clinical and endoscopic outcomes of vedolizumab in Chinese patients with ulcerative colitis in a real-world clinical practice setting, and to identify factors associated with treatment response and drug persistence.

## 2 Methods

### 2.1 Study design and patient population

This single-center, retrospective, descriptive cohort study was conducted at The First Affiliated Hospital of Anhui Medical University, a tertiary medical institution located in Hefei City, Anhui Province, People’s Republic of China. The study was approved by the Clinical Medical Research Ethics Committee of the First Affiliated Hospital of Anhui Medical University (approval number: PJ 2025-11-03). The need for individual patient consent was waived by the ethics committee due to the retrospective nature of the study, and all patient data were fully de-identified prior to analysis. The study was conducted in accordance with the principles of the Declaration of Helsinki.

The study enrolled adults aged ≥18 years who received their first vedolizumab dose for UC treatment between December 2020 and August 2023. Inclusion criteria were: (1) confirmed diagnosis of ulcerative colitis based on standard clinical, endoscopic, and histological criteria; (2) age ≥18 years; (3) first-time treatment with vedolizumab during the study period; (4) completed at least 24 months of follow-up or discontinued treatment earlier due to any reason. Exclusion criteria were: (1) surgical interventions (e.g., total colectomy, ileostomy) before VDZ induction; (2) active participation in any interventional clinical trial during the study period; (3) pregnancy or lactation at the time of VDZ initiation. For the benefit of international readers, it should be noted that vedolizumab has been included in China’s National Reimbursement Drug List since 2020, making it accessible to eligible patients through national health insurance coverage, thereby substantially reducing the financial barrier to treatment.

### 2.2 Data collection and variables

Baseline data were collected by chart review including demographics, disease duration and extent at time of diagnosis (using the Montreal classification for UC), current and previous IBD therapies, and disease activity parameters at baseline, if available. In addition, VDZ dosing, dose optimization requirements, drug persistence, discontinuation reasons and IBD-related hospitalizations were recorded. Concomitant therapies comprising 5-ASA (oral or topical), corticosteroids, and immunomodulators were allowed during the course of the study and noted for all participants.

### 2.3 Treatment protocol

Vedolizumab (Entyvio®, Takeda Pharmaceutical Company Limited, Osaka, Japan; imported and distributed by Takeda (China) Holdings Co., Ltd.) was administered intravenously according to standard induction (300 mg at weeks 0, 2, and 6) followed by maintenance therapy (300 mg every 8 weeks) (4). As the subcutaneous formulation was not yet widely available in China during the study period, all patients received the intravenous formulation. Dose optimization (interval shortening to 4–6 weeks) was permitted according to physician discretion. The choice of the biologic was guided primarily by physicians’ personal confidence and belief about the efficacy and safety of the agent.

### 2.4 Outcome and definitions

According to the Montreal classification (10), UC was classified as proctitis (E1), left-sided UC (E2), and total colitis (E3). Classification of disease activity as mild (3–5 points), moderate (6–10 points) and severe (11–12 points) was based on the total Mayo score, which includes stool frequency, rectal bleeding, Mayo endoscopy score (MES), and overall assessment by the physician. The partial Mayo score (PMS) was defined as the Mayo score without MES. PMS was assessed before the induction and before each infusion. Clinical response was defined as a decrease of at least 2 points from baseline in PMS (or at least 30% in PMS) accompanied by an absolute score for rectal bleeding of ≤ 1 or a decrease of at least one point from baseline. Clinical remission was defined as PMS ≤ 2 with no subscore >1 and rectal bleeding score = 0. Endoscopic remission was defined as MES ≤ 1.

The primary outcome of this study was VDZ persistence. The primary endpoint was clinical remission at week 104, defined as PMS ≤ 2 with no subscore >1 and rectal bleeding score = 0. Secondary outcomes included clinical response at weeks 14, 52, 78, and 104, clinical remission at weeks 14, 52, and 78, endoscopic remission at weeks 52 and 104, baseline predictors of VDZ persistence and serious adverse events. An adverse drug reaction (ADR) was defined as any unwanted or harmful reaction experienced by a patient following vedolizumab administration during study follow-up that was documented by the treating physician as possibly, probably, or definitely related to treatment. End of follow-up was defined as either discontinuation, loss to follow-up, or the end of the data collection period. Follow-up time was calculated from the initial induction date until end of follow-up.

### 2.5 Follow-up

The end of the FU was set at week 104. The mean follow up was calculated by including patients who discontinued treatment prior to week 104 due to primary non-response, secondary loss of response, ADRs, or the need for surgery. The PMS was calculated at each infusion. In the case of non-response or worsening of clinical condition, endoscopic activity reassessed by recto-sigmoidoscopy when considered appropriate. Steroids or immunosuppressants were added when deemed necessary by the physician during the FU. The need for dose optimization or drug switch was also reported.

### 2.6 Statistical analysis

All statistical analyses were performed using SPSS (version 24.0, IBM Corp., Armonk, NY, USA) and GraphPad Prism (version 10.1.2, GraphPad Software, San Diego, CA, USA).

For descriptive statistics, categorical variables were presented as counts and percentages. Continuous variables were tested for normality using the Shapiro–Wilk test. Normally distributed data were reported as mean ± standard deviation (SD), while non-normally distributed data were presented as median with interquartile range (IQR).

For comparative analyses, independent *t*-tests were used for normally distributed continuous variables when comparing two groups, and Mann–Whitney U tests were used for non-normally distributed variables. For comparisons among multiple groups, one-way ANOVA with post-hoc tests or Kruskal-Wallis tests with Bonferroni adjustment for multiple comparisons were applied as appropriate. Paired observations were evaluated using paired *t*-tests or Wilcoxon signed-rank tests.

Regarding the handling of missing data and treatment discontinuations, patients lost to follow-up before week 52, 78, and 104 with unknown treatment outcomes were excluded from remission rate calculations. Patients who discontinued treatment during follow-up due to inadequate efficacy or adverse events were classified as negative outcomes when calculating clinical and endoscopic remission rates, in accordance with the intention-to-treat principle. For partial Mayo score and endoscopic Mayo subscore, no imputation was performed for missing data at each follow-up time point. When comparing changes over time, only patients with available data at both baseline and the corresponding follow-up time point were included in paired analyses.

For regression analyses, univariate logistic regression was used to identify potential factors (*p* < 0.10) associated with endoscopic remission at week 52. Variables with *p* < 0.05 in univariate analysis were included in multivariate models using forward stepwise selection to determine independent predictors. Multicollinearity was assessed using variance inflation factor, with a value greater than 5 indicating multicollinearity. Odds ratios with 95% confidence intervals were reported.

For survival analysis, Kaplan–Meier survival analysis was used to evaluate drug persistence over time, with group comparisons using the log-rank test. The event was defined as drug discontinuation. Patients lost to follow-up or remaining on treatment at study endpoint were censored. Univariate and multivariable analyses of factors associated with vedolizumab persistence were performed using Cox proportional hazards regression models, with variables showing *p* < 0.05 in univariate analysis included in multivariate models. Hazard ratios and 95% confidence intervals were calculated.

Regarding the significance level, a two-tailed *p*-value < 0.05 was considered statistically significant. Given the exploratory nature of this real-world study, no adjustments were made for multiple comparisons, which should be considered when interpreting the results.

### 2.7 Ethical considerations

This study was approved by the Clinical Medical Research Ethics Committee of the First Affiliated Hospital of Anhui Medical University (approval number: PJ 2025-11-03). The study utilized anonymized clinical data collected during routine care without additional interventions. The need for individual patient consent was waived by the ethics committee due to the retrospective nature of the study, and all patient data were fully de-identified prior to analysis. The study adhered to the principles of the Declaration of Helsinki (1964 and subsequent amendments).

## 3 Results

### 3.1 Patient characteristics

**Figure 1:**
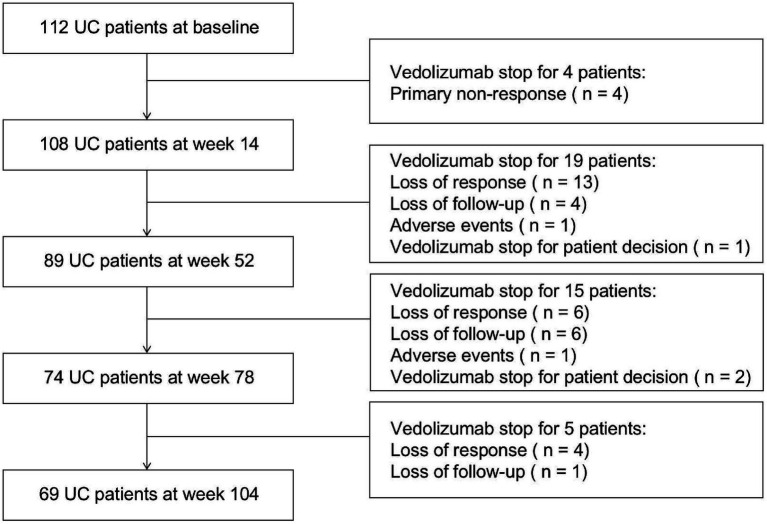
Flow-chart of patient inclusion.

A total of 119 UC patients were included in the study with a median duration of 32.5 months (IQR, 14.0–47.8 months) on VDZ. Among them, 5 patients were lost to follow-up, and 2 discontinued treatment for personal reasons unrelated to efficacy or safety. These 7 patients were excluded from the efficacy analysis due to the lack of evaluable outcomes. The remaining 112 patients were included in the final analysis cohort. Of these, 4 patients discontinued treatment due to primary non-response. A total of 69 patients completed at least 104 weeks of follow-up (Figure 1). Baseline demographic and clinical characteristics of the 112 analyzed patients are summarized in Table 1. Baseline endoscopy was performed in all 112 patients of the cohort, with 90 individuals demonstrating endoscopic activity at week 52 and 54 individuals demonstrating endoscopic activity at week 104.

**Table 1:** Baseline demographic and clinical characteristics of the study population.

Variables	*N* = 112
Sex, *n* (%)^a^	
Male	62 (55.36)
Female	50 (44.64)
Age, years^b^	40.37 ± 13.57
BMI, kg/m^2^^c^	20.51 (19.05, 23.17)
Smoking, *n* (%)^a^	
Yes	20 (17.86)
No	92 (82.14)
Disease duration, months^c^	45.50 (18.75, 90.25)
Age at diagnosis, years^c^	33 (25, 45)
History of surgery, *n* (%)^a^	
Yes	4 (3.57)
No	108 (96.43)
Extra-intestinal manifestations, *n* (%)^a^	
Yes	9 (8.04)
No	103 (91.96)
Disease extent, *n* (%)^a^	
Proctitis	0 (0.00)
Left-sided colitis	44 (39.29)
Extensive colitis	68 (60.71)
Disease activity, *n* (%)^a^	
Mild	15 (13.39)
Moderate	89 (79.46)
Severe	8 (7.14)
WBC, ×10^9^/L^c^	6.70 (5.27, 8.40)
Hemoglobin, g/L^c^	117.00 (95.50, 133.00)
Platelet count, ×10^9^/L^c^	256.00 (203.50, 344.25)
Albumin, g/L^b^	37.99 ± 4.84
CRP, mg/L^c^	3.45 (1.10, 10.45)
FCP, ug/g^c^	1013.00 (532.50, 1399.25)
Partial Mayo score^c^	6.00 (4.00, 6.00)
Endoscopic Mayo subscore^c^	3.00 (2.00, 3.00)
Total Mayo score^c^	8.00 (6.00, 9.00)
Previous 5-ASA therapy, *n* (%)^a^	
Yes	109 (97.32)
No	3 (2.68)
Previous steroid therapy, *n* (%)^a^	
Yes	47 (41.96)
No	65 (58.04)
Previous azathioprine therapy, *n* (%)^a^	
Yes	6 (5.36)
No	106 (94.64)
Any previous biologics exposure, *n* (%)^a^	
Yes	16 (14.29)
No	96 (85.71)
Concomitant medication, *n* (%)^a^	
None	35 (31.25)
5-ASA	42 (37.50)
Steroid	3 (2.68)
5-ASA and steroid	32 (28.57)
Azathioprine	0 (0.00)
Biologics	0 (0.00)

BMI, body mass index; WBC, white blood cell count; CRP, C-reactive protein; FCP, faecal calprotectin; 5-ASA, 5-aminosalicylic acid.

a Qualitative variable, *n* (%).

b Normal quantitative variable, mean (±SD).

c Non-normal quantitative variable, median (interquartile range).

Mean age of the cohort patients was 40.37 ± 13.57 years and median disease duration was 45.50 months (IQR 18.75–90.25). All 112 patients were of Han Chinese ethnicity, reflecting the predominant ethnic composition of the region. Disease distribution included extensive colitis (60.71%), left-sided colitis (39.29%), and no proctitis cases. Most patients (79.46%) demonstrated moderate disease activity at baseline. Of 112 patients, 109 (97.32%) patients had a prior treatment with 5-ASA, 47/112 (41.96%) with corticosteroids, and 6/112 (5.36%) with azathioprine. Overall 77/112 (68.75%) patients had a history of concomitant treatment at inclusion; 42/112 (37.50%) patients were on concomitant treatment with 5-ASA; 3/112 (2.68%) patients were on concomitant treatment with oral corticosteroids; 32/112 (28.57%) were on concomitant treatment with both 5-ASA and oral corticosteroids. Of the 112 patients, 96 (85.71%) were biologic-naïve at baseline. The remaining 16 (14.29%) had a history of biologic therapy: 14 with infliximab, one with infliximab and tofacitinib, and one with infliximab and guselkumab. The main reasons for discontinuing the biologic therapy was non-response (14/16; 87.50%) and only a minority was due to intolerance (2/16; 12.50%).

### 3.2 Clinical effectiveness

**Figure 2:**
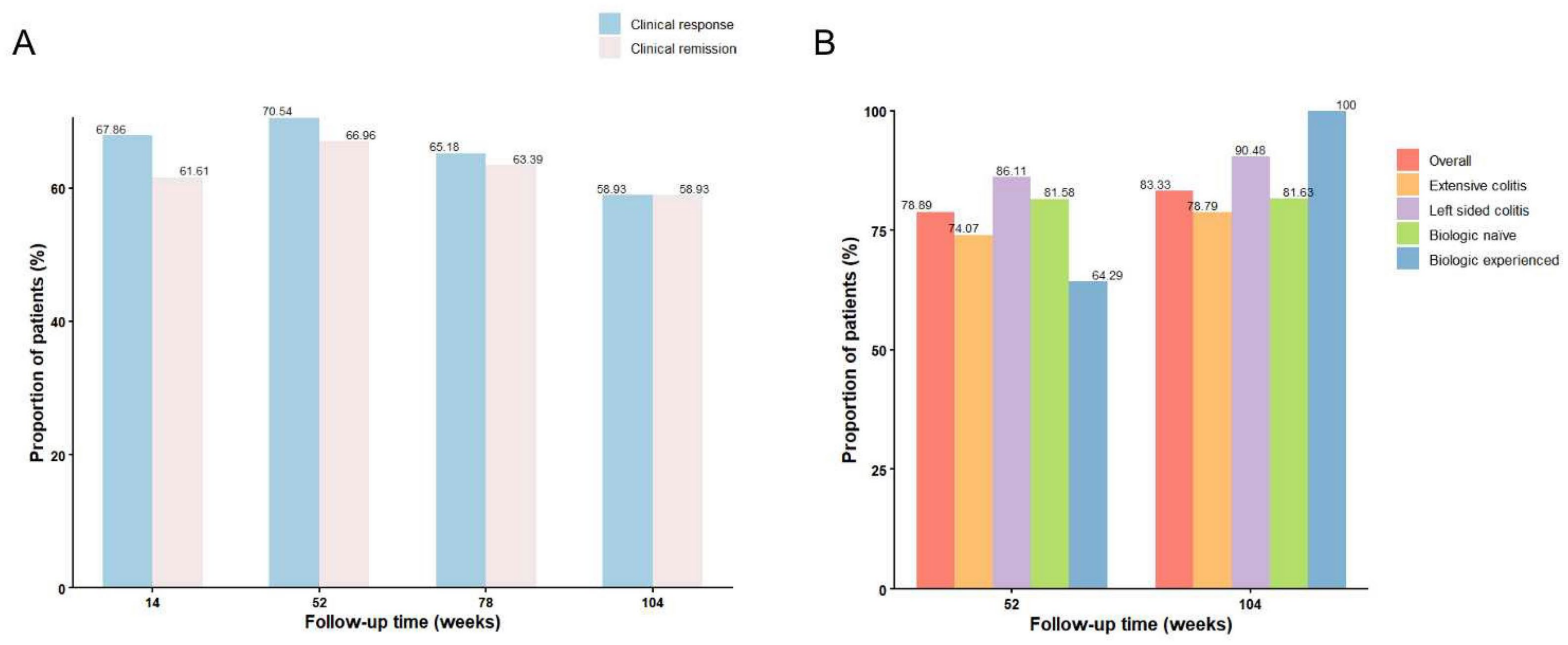
Efficacy outcomes over time. **(A)** Clinical response and clinical remission rates at weeks 14, 52, 78, and 104. **(B)** Rates of complete mucosal healing at week 52 and 104, analyzed separately for patients with extensive or left-sided colitis, and for those who were biologic-naïve or biologic-experienced.

Overall, 76 (67.86%) and 69 (61.61%) of the 112 patients achieved clinical response and clinical remission at week 14, respectively. By week 52, the rates for clinical response, clinical remission and steroid-free clinical remission were 70.54% (79/112), 66.96% (75/112) and 66.07% (74/112), respectively. These rates were 65.18% (73/112), 63.39% (71/112) and 63.39% (71/112) at week 78, and 58.93% (66/112), 58.93% (66/112) and 58.04% (65/112) at week 104 (Figure 2A).

Among 96 patients with VDZ as a first-line biologic, 67 (69.79%) achieving clinical response and 58 (60.42%) achieving clinical remission at week 14. By week 52, the rates for clinical response, clinical remission and steroid-free clinical remission were 70.83% (68/96), 66.67% (64/96) and 65.63% (63/96), respectively. These rates were 66.67% (64/96), 65.63% (63/96), 65.63% (63/96) at week 78, and 60.42% (58/96), 60.42% (58/96), and 60.42% (58/96) at week 104. In contrast, in the 16 patients with prior biologic exposure, 11 (68.75%) and 9 (56.25%) attained clinical response and remission at week 14, respectively. At week 52, 11 patients (68.75%) met all three endpoints (steroid-free clinical remission, clinical remission, and clinical response). However, these rates decreased to 50.0–56.25% at week 78 and to 50.0% across all endpoints by week 104. No statistically significant differences in response or remission rates were observed between the biologic-naïve and biologic-experienced groups at any time point (*p* > 0.05).

### 3.3 Mucosal healing

At baseline, all 112 patients in the cohort underwent endoscopy. The distribution of Mayo endoscopic subscore (MES) was as follows: 57 patients (50.9%) had MES 3, among whom 41 had extensive colitis and 16 had left-sided colitis; 54 patients (48.2%) had MES 2; and 1 patient (0.9%) had MES 1. At week 52, 90 patients completed follow-up endoscopy, of whom 71 achieved mucosal healing (defined as MES ≤ 1). Mucosal healing rates were 74.07% (40/54) in patients with extensive colitis and 86.11% (31/36) in those with left-sided colitis. According to prior biologic exposure, mucosal healing rates were 81.58% (62/76) in biologic-naïve patients and 64.29% (9/14) in biologic-experienced patients.

At week 104, 54 patients completed follow-up endoscopy, of whom 45 achieved mucosal healing. Mucosal healing rates by disease extent were 78.79% (26/33) in patients with extensive colitis and 90.48% (19/21) in those with left-sided colitis. Among biologic-naïve patients, the mucosal healing rate was 81.63% (40/49). Notably, all five biologic-experienced patients who underwent endoscopy at week 104 achieved mucosal healing (Figure 2B).

### 3.4 VDZ persistence

**Figure 3:**
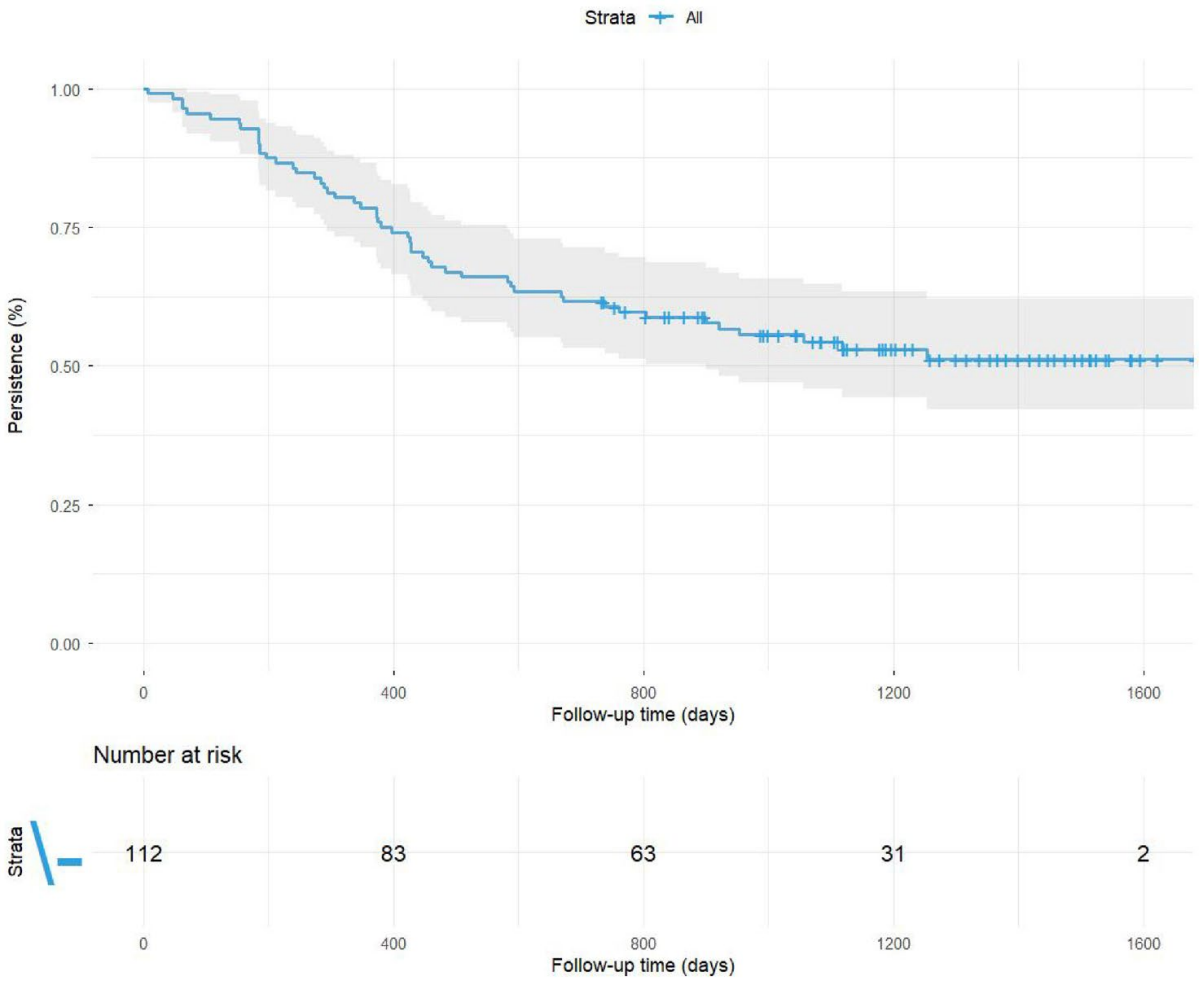
Cumulative persistence rate of VDZ.

Median time on VDZ of 32.5 months (IQR, 14.0–47.8 months). At the end of follow-up, a total of n = 60/112 (53.57%) patients remained on VDZ. VDZ persistence at 1 year was 78.57% (88/112), 61.61% (69/112) at 2 years (Figure 3). Among all 112 patients, 44 had completed at least 3 years of follow-up and 15 had completed at least 4 years. The median time to VDZ discontinuation was 13.5 months (IQR, 7.9–22.0 months). Reasons for drug discontinuation included the following: PNR: 6.25% (7), secondary loss of response: 18.75% (21), adverse events: 1.79% (2), long-term remission: 8.04% (9) and other reasons: 12.50% (14) (patient’s decision *n* = 10, need of drug change to treat another autoimmune disorder *n* = 2, lost follow-up *n* = 2).

A total of 9 (8.04%) patients needed VDZ intensification to every 4 or 6 weeks, with a median time to drug intensification of 4.17 months. Of these patients, 9 (88.89%) discontinued VDZ during follow-up and only 1 (11.11%) remained on it at the end of the follow-up.

### 3.5 Predictors of drug persistence

**Figure 4:**
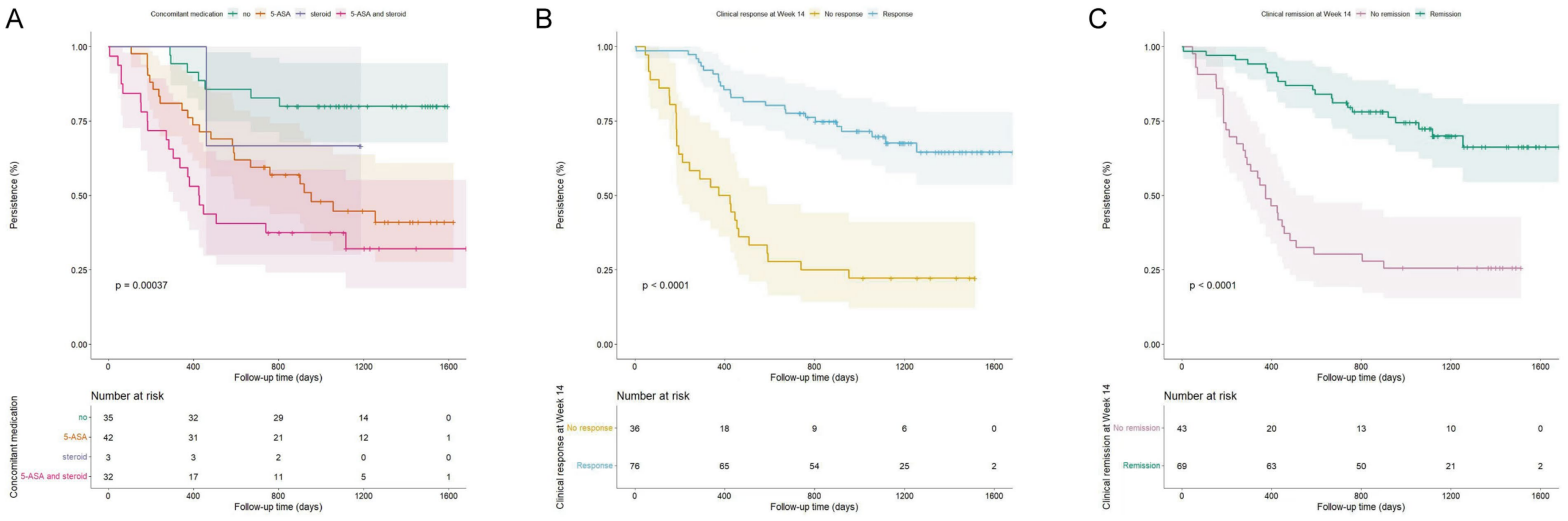
Kaplan–Meier analysis of vedolizumab treatment persistence. The cumulative probability of treatment persistence is shown, stratified by three baseline factors. **(A)** Concomitant medication use at initiation. **(B)** Clinical response status at Week 14. **(C)** Clinical remission status at Week 14. Differences between groups were assessed using the log-rank test. Shaded areas represent 95% confidence intervals. Corresponding hazard ratios (HR) and 95% confidence intervals (CI) from the multivariable Cox regression model are presented in Table 2.

The results of the multivariable Cox regression analysis identified several factors that significantly influenced vedolizumab drug persistence. Notably, the use of concomitant medications at baseline was associated with a higher risk of treatment discontinuation, particularly for patients receiving 5-ASA alone (HR = 3.13, 95% CI 1.34–7.33, *p* = 0.008) and for those receiving combined 5-ASA and corticosteroid therapy (HR = 5.03, 95% CI 2.10–12.05, *p* < 0.001) (Figure 4A). Conversely, achieving clinical response (HR = 0.38, 95% CI 0.21–0.70, *p* = 0.002) (Figure 4B) or clinical remission (HR = 0.33, 95% CI 0.17–0.62, *p* < 0.001) (Figure 4C) at Week 14 emerged as strong predictors of superior drug persistence over the 2-year follow-up period (Table 2).

**Table 2:** Predictors of vedolizumab treatment persistence: univariate and multivariate Cox regression analysis.

Variable	Univariable regression			Multivariable regression		
	HR	95% CI	*p*-value	HR	95% CI	*p*-value
Sex						
Male	Ref					
Female	0.937	0.542–1.620	0.815			
Age	0.993	0.973–1.014	0.520			
BMI	0.925	0.848–1.008	0.077			
Smoking						
No	Ref					
Yes	0.652	0.294–1.445	0.292			
History of surgery						
No	Ref					
Yes	1.040	0.253–4.277	0.956			
EIMS						
No	Ref					
Yes	1.609	0.687–3.769	0.273			
Disease duration	1.000	0.996–3.769	0.972			
Age at diagnosis	0.993	0.971–1.014	0.507			
Disease extent						
Left-sided colitis	Ref					
Extensive colitis	1.097	0.628–1.919	0.745			
Previous 5-ASA therapy						
No	Ref					
Yes	1.699	0.235–12.299	0.600			
Previous steroid therapy						
No	Ref					
Yes	1.426	0.827–2.460	0.201			
Previous azathioprine therapy						
No	Ref					
Yes	1.036	0.323–3.327	0.953			
Any previous biologics exposure						
No	Ref					
Yes	1.119	0.527–2.379	0.769			
WBC	1.036	0.950–1.130	0.422			
HB	0.998	0.987–1.009	0.694			
PLT	1.000	0.998–1.002	0.983			
CRP	1.003	0.992–1.013	0.626			
ALB	0.989	0.936–1.045	0.701			
FCP	1.000	0.999–1.000	0.168			
Endoscopic Mayo subscore	1.626	0.940–2.815	0.082			
Partial Mayo score	1.178	0.966–1.437	0.106			
Total Mayo score	1.166	0.990–1.373	0.065			
Disease activity						
Mild	Ref					
Moderate	1.308	0.557–3.073	0.538			
Severe	0.956	0.239–3.825	0.950			
Concomitant medication						
No	Ref					
5-ASA	3.381	1.449–7.887	0.005	3.133	1.339–7.331	0.008
Steroid	1.733	0.213–14.097	0.607			
5-ASA and steroid	5.491	2.326–12.959	<0.001	5.034	2.103–12.050	<0.001
Clinical response at Week 14						
No						
Yes	0.232	0.134–0.402	<0.001	0.382	0.207–0.704	0.002
Clinical remission at Week 14						
No						
Yes	0.226	0.129–0.399	<0.001	0.327	0.172–0.619	<0.001

Results from Cox proportional hazards models for drug persistence. Concomitant use of 5-ASA or 5-ASA + steroid was associated with poorer drug persistence (higher risk of discontinuation), while clinical response or remission at Week 14 was associated with improved persistence. HR, Hazard Ratio; CI, Confidence Interval; HB, Hemoglobin; PLT, Platelet count; ALB, Albumin.

### 3.6 Endoscopic remission predictors

Multivariable logistic regression analysis was performed to identify baseline and early treatment factors predictive of endoscopic remission (Mayo endoscopic subscore ≤ 1) at Week 52. The model demonstrated that achieving clinical remission by Week 14 was the strongest independent predictor, significantly associated with a more than six-fold increased likelihood of attaining endoscopic remission at 1 year (OR = 6.07, 95% CI 2.11–17.41, *p* < 0.001). In contrast, the concomitant use of both 5-ASA and corticosteroids at the initiation of vedolizumab therapy was associated with a significantly lower probability of achieving endoscopic remission (OR = 0.22, 95% CI 0.05–0.91, *p* = 0.037) (Table 3).

**Table 3:** Predictors of endoscopic remission at week 52: univariate and multivariate logistic regression analysis.

Variable	Univariable regression			Multivariable regression		
	OR	95% CI	*p*-value	OR	95% CI	*p*-value
Clinical remission at week 14						
No	Ref					
Yes	10.962	4.592–28.055	<0.001	6.067	2.114–17.412	<0.001
Clinical response at week 14						
No	Ref					
Yes	9.750	4.030–25.355	<0.001	5.639	1.852–17.172	0.002
Concomitant medication						
No	Ref					
5-ASA	0.336	0.107–0.950	0.047			
Steroid	0.414	0.034–9.740	0.499			
5-ASA and steroid	0.142	0.043–0.417	0.001	0.220	0.053–0.912	0.0369
Previous steroid therapy						
No	Ref					
Yes	0.431	0.195–0.937	0.035			
BMI	1.126	0.993–1.289	0.073			
Smoking						
No	Ref					
Yes	2.815	0.945–10.418	0.083			
Disease extent						
Left-sided colitis	Ref					
Extensive colitis	0.564	0.247–1.248	0.164			
Partial Mayo score	0.841	0.636–1.102	0.213			
Total Mayo score	0.877	0.697–1.096	0.254			
ALB	1.036	0.957–1.124	0.381			
Disease duration	0.998	0.992–1.004	0.439			
Previous azathioprine therapy						
No	Ref					
Yes	0.582	0.103–3.277	0.520			
FCP	1.000	1.000–1.001	0.562			
Any previous biologics exposure						
No	Ref					
Yes	0.738	0.253–2.227	0.578			
History of surgery						
No	Ref					
Yes	0.588	0.068–5.057	0.603			
EIMS						
No	Ref					
Yes	0.731	0.183–3.108	0.655			
Disease activity						
Mild	Ref					
Moderate	0.771	0.224–2.366	0.660			
Severe	1.500	0.233–12.859	0.680			
Endoscopic Mayo subscore	0.867	0.410–1.817	0.706			
HB	1.003	0.987–1.019	0.713			
PLT	1.001	0.997–1.004	0.750			
Age at diagnosis	1.004	0.974–1.035	0.791			
CRP	1.002	0.985–1.021	0.842			
Sex						
Female	Ref					
Male	0.962	0.446–2.087	0.922			
Age	0.999	0.971–1.028	0.947			
WBC	0.996	0.871–1.146	0.957			
Previous 5-ASA therapy						
No	Ref					
Yes	<0.001	NA–∞	0.994			

Logistic regression analysis identifying factors associated with endoscopic remission at Week 52. Clinical remission and clinical response at Week 14 were significant predictors. OR, Odds Ratio; CI, Confidence Interval.

### 3.7 Safety profile

A total of 9 patients experienced ADRs, corresponding to an incidence rate of 3.8 events per 100 patient-years of follow-up. Among these, 1 patient presented with a skin reaction, 4 reported mild infectious symptoms, and 2 experienced arthralgia. Additionally, 2 patients required UC-related surgical intervention due to disease progression; both recovered well postoperatively. No serious infections or treatment-related severe adverse events were reported throughout the study period.

## 4 Discussion

This real-world cohort study provides comprehensive, long-term evidence on the effectiveness and durability of vedolizumab (VDZ) in Chinese patients with ulcerative colitis (UC), with sustained clinical and endoscopic outcomes documented through 104 weeks of follow-up.

The findings demonstrate that VDZ can induce and maintain high rates of clinical remission in a real-world setting. The clinical remission rate in our cohort reached 66.96% at week 52, which is notably higher than the 44.3% reported in the pivotal GEMINI 1 trial at the same time point (4). This difference aligns with findings from several large-scale real-world cohorts, such as the US VICTORY consortium study, which reported 52-week clinical remission rates of approximately 60% in UC patients (11), and the European EVOLVE study, which observed similar real-world effectiveness exceeding that of RCT settings (12). The flexibility in dosing, treatment continuation beyond standard induction in partial responders, and individualized concomitant therapy strategies in clinical practice likely contribute to these enhanced outcomes (13). Furthermore, the 58.93% remission rate at week 104 reinforces the long-term sustainability of VDZ’s effect. This is consistent with the GEMINI long-term extension (LTS) study, which reported sustained clinical remission in 42.2% of UC patients at week 152 (6), and with recent real-world data from the UK IBD Registry showing continued effectiveness beyond 2 years (14).

Importantly, the mucosal healing rates observed in our study—79.17% at week 52 and 83.33% at week 104—highlight VDZ’s capacity to achieve deep remission, a treatment goal strongly associated with improved long-term outcomes, including reduced hospitalization and colectomy risk (15, 16). These rates are comparable to or exceed those reported in other real-world cohorts, such as the Italian IBD Group study (mucosal healing in 71.4% at 52 weeks) (17) and the Spanish ENEIDA registry (76.5% at 1 year) (18). The progressive improvement in endoscopic outcomes over time underscores VDZ’s characteristically delayed mechanism of action. This supports current international consensus guidelines recommending against premature discontinuation before completing at least 14 weeks of therapy, as endoscopic response often continues to evolve beyond the induction phase (19, 20).

In comparison with other biologic classes, network meta-analyses have positioned VDZ as having comparable long-term efficacy to anti-TNF agents in maintaining remission, particularly in bio-naïve patients, while offering a potentially superior safety profile regarding serious infections (21, 22). Recent comparative effectiveness studies also suggest that VDZ may be associated with higher drug persistence than some anti-TNF agents over 24 months (23). The data thus contribute robust, long-term, real-world evidence from an Asian population to the global literature, reinforcing VDZ as a durable and effective therapeutic option for maintaining both clinical and endoscopic remission in UC.

A central finding of our analysis is the notable trend toward superior clinical outcomes in biologic-naïve patients compared to those with prior anti-TNF exposure, though statistical significance was not reached, likely due to the limited sample size of the biologic-experienced subgroup. Numerically, biologic-naïve patients maintained higher remission rates at all time points, with 60.42% achieving clinical remission at week 104, compared to 50.0% in the biologic-experienced group. This disparity aligns with accumulating real-world evidence suggesting that VDZ may be most effective when positioned earlier in the treatment sequence. In the VICTORY consortium study, clinical remission rates at week 52 were significantly higher in anti-TNF–naïve UC patients (58.9%) compared to anti-TNF–exposed patients (43.3%) (11). Similarly, the EVOLVE study reported superior mucosal healing rates in biologic-naïve patients after 52 weeks of VDZ therapy (12). The diminished response in patients with prior biologic failure may reflect a more refractory disease phenotype, potential immunogenic alterations following anti-TNF exposure, or differences in underlying inflammatory pathways (24), and also the fact that biologic-naïve patients generally have shorter disease duration and lower cumulative inflammatory burden. These observations carry important implications for therapeutic sequencing in UC management, supporting the consideration of VDZ as a first-line biologic agent, particularly in regions where tuberculosis and other opportunistic infections are prevalent, given its favorable gut-selective safety profile.

This study identifies early clinical response at week 14 as a robust and practical predictor of long-term treatment success. Patients achieving clinical remission or response at this time point demonstrated significantly superior drug persistence and were over six times more likely to attain endoscopic remission at 1 year (OR = 6.07, *p* < 0.001). These results are strongly supported by recent evidence highlighting the prognostic value of early response assessment in biologic therapy for UC. A post-hoc analysis of the GEMINI 1 trial demonstrated that clinical response at week 14 was associated with significantly higher rates of sustained remission and mucosal healing at week 52 (25). Similarly, the VISIBLE 1 trial reaffirmed that early response to vedolizumab—whether assessed clinically or via fecal calprotectin—correlates strongly with long-term endoscopic outcomes (7). Beyond VDZ, a systematic review concluded that week 14 response is a consistently meaningful predictor of subsequent remission across multiple biologic classes in UC, supporting its utility as a decision point in treat-to-target strategies (26). This finding provides clinicians with a valuable tool for dynamic treatment decision-making. Regular assessment at the end of the induction phase allows for the early identification of primary non-responders, enabling a timely transition to alternative therapeutic strategies, such as dose intensification, switching to another mechanism of action, or considering combination therapy—an approach emphasized in the recent ECCO-ESGAR guideline on therapeutic optimization in IBD (20). Conversely, confirmation of early response reinforces continued treatment with VDZ, optimizing patient adherence and healthcare resource allocation. This predictive model enhances personalized medicine in UC, moving toward a treat-to-target approach guided by early objective milestones.

An intriguing and clinically relevant finding was the independent association between concomitant medication use at baseline—particularly the combination of 5-ASA and corticosteroids—and poorer long-term outcomes. This association likely does not indicate a detrimental pharmacological interaction, but rather serves as a proxy for greater disease severity, steroid dependence, or a more complex clinical phenotype at treatment initiation. Furthermore, in real-world settings, oral corticosteroids, being inexpensive and readily accessible, may be used not only for acute flare control but also influenced by physician prescribing habits and economic considerations, leading to broader or prolonged use compared to strictly protocol-driven RCTs, thereby introducing confounding bias. Recent evidence further contextualizes this finding. A large-scale retrospective cohort study from the French nationwide health database (EPIMAD) demonstrated that concomitant corticosteroid use at biologic initiation was strongly associated with increased risk of treatment failure and surgery in UC patients, independent of the biologic class used (27). Similarly, a post-hoc analysis of the VARSITY trial data indicated that patients receiving vedolizumab while on baseline corticosteroids had lower rates of clinical remission and mucosal healing compared to those not on steroids, reinforcing steroids as a marker of more difficult-to-treat disease rather than a direct cause of biologic inefficacy (28). The specific combination of 5-ASA and steroids may identify a distinct phenotypic subgroup, as suggested by a multicenter Italian study which found that this combination at baseline correlated with higher baseline inflammatory burden and poorer long-term outcomes even after adjusting for disease activity scores (29).

To test this hypothesis, a post-hoc analysis was performed comparing baseline disease characteristics between patients receiving concomitant 5-ASA and corticosteroids (*n* = 32) and those without any concomitant medication (*n* = 38). Patients in the concomitant medication group exhibited numerically higher disease activity across all parameters, including partial Mayo score (6.0 vs. 5.0), endoscopic Mayo subscore (3.0 vs. 2.0), disease duration (48.0 vs. 36.0 months), CRP (4.2 vs. 2.8 mg/L), and fecal calprotectin (1,120 vs. 856 μg/g). Although these differences did not reach statistical significance—likely due to the limited sample size—the consistent trend across multiple parameters supports the interpretation that concomitant medication use serves as a clinical proxy for greater disease burden rather than a direct pharmacological interaction affecting VDZ efficacy.

Importantly, this observation suggests that for a significant proportion of VDZ responders, long-term combination with conventional immunomodulators may be unnecessary, allowing for treatment simplification, reduction of polypharmacy, and minimization of cumulative steroid exposure. The gut-selective mechanism of VDZ appears to provide sufficient immunosuppression for many patients, potentially obviating the need for additional systemic agents. This concept is supported by a 2023 systematic review and meta-analysis focusing on vedolizumab monotherapy versus combination therapy, which concluded that the addition of immunomodulators did not significantly enhance efficacy in vedolizumab-treated UC patients, while increasing the risk of adverse events (30). Furthermore, a prospective de-escalation study specifically in vedolizumab-treated patients showed that withdrawal of concomitant immunomodulators after 6 months of sustained remission was successful in over 80% of cases without relapse at 1 year (31).

The safety profile observed in this two-year cohort further consolidates the favorable risk–benefit ratio of VDZ. With an ADR incidence of only 3.8 per 100 patient-years and no reports of serious infections or treatment-related severe adverse events, VDZ demonstrates a safety advantage consistent with its localized mechanism of action. Recent meta-analyses of real-world and trial data consistently support this favorable profile. A 2023 pooled analysis of global safety databases concluded that vedolizumab was associated with significantly lower rates of serious infections and opportunistic infections compared to anti-TNF agents in IBD patients (32). Specifically in elderly populations (≥60 years), a large multicenter cohort study demonstrated that vedolizumab had a comparable efficacy but a significantly lower risk of serious infections than anti-TNF therapies, making it particularly suitable for this vulnerable group (33–35). This safety differential should be actively considered in shared decision-making, particularly when comparing VDZ to agents with broader immunosuppressive effects, such as anti-TNF therapies or JAK inhibitors. Current international guidelines reflect this evidence, with the 2023 ACG clinical guideline explicitly recommending vedolizumab over anti-TNF agents for UC patients with significant infection risk factors due to its superior safety profile (36).

While the findings align with and extend the global real-world evidence on VDZ, several limitations warrant consideration. The retrospective, single-center design introduces potential selection bias and may limit the generalizability of these results to broader UC populations, including those with different genetic backgrounds or healthcare access. The absence of therapeutic drug monitoring data represents another constraint, as drug trough levels and anti-drug antibodies could provide mechanistic insights into secondary loss of response and guide dose optimization strategies. In particular, the modest sample size, especially in the biologic-experienced subgroup, limited statistical power and constrained our ability to draw definitive conclusions from subgroup comparisons. The absence of statistical significance for numerically superior outcomes in biologic-naïve patients likely reflects insufficient power rather than equivalence. These limitations should be considered when interpreting the findings, which warrant validation in larger, prospective cohorts. Furthermore, as is typical of retrospective real-world studies, the safety data are limited to adverse drug reactions documented in the medical records. Mild or unrelated adverse events may have been underreported by treating physicians, potentially leading to an underestimation of the overall adverse event burden. This limitation should be considered when interpreting the favorable safety profile reported in this study. Moreover, the flexible endoscopic follow-up schedule, though reflective of real-world practice, introduces variability in the precise assessment of mucosal healing timelines. Despite these limitations, the extended follow-up duration, comprehensive outcome assessment, and inclusion of a heterogeneous patient population enhance the ecological validity of our findings. Future prospective studies incorporating biomarker-driven stratification, head-to-head comparisons with newer drug classes, and health-economic evaluations will further refine the positioning of VDZ within the evolving UC treatment paradigm.

## 5 Conclusion

In conclusion, this real-world study confirms the sustained effectiveness and favorable safety profile of vedolizumab in Chinese patients with ulcerative colitis over a 2-year period. Early clinical response is a strong predictor of long-term treatment success and endoscopic healing. Concomitant use of corticosteroids with 5-ASA at baseline may signal a more refractory disease course and reduced VDZ persistence. These findings support the integration of early response assessment into clinical decision-making and underscore VDZ’s role as a durable and well-tolerated therapeutic option in real-world practice. However, the retrospective, single-center design and modest sample size, particularly for subgroup analyses, represent limitations that should be considered when interpreting these results, and the findings warrant validation in larger, prospective multicenter studies.

## Data Availability

The original contributions presented in the study are included in the article/supplementary material, further inquiries can be directed to the corresponding author.
